# Rational Design of T Cell Receptors with Enhanced Sensitivity for Antigen

**DOI:** 10.1371/journal.pone.0018027

**Published:** 2011-03-23

**Authors:** Rajshekhar Alli, Ziwei M. Zhang, Phuong Nguyen, Jie J. Zheng, Terrence L. Geiger

**Affiliations:** 1 Department of Pathology, St. Jude Children's Research Hospital, Memphis, Tennessee, United States of America; 2 Department of Structural Biology, St. Jude Children's Research Hospital, Memphis, Tennessee, United States of America; 3 Department of Pathology, University of Tennessee Health Sciences Center, Memphis, Tennessee, United States of America; New York University, United States of America

## Abstract

Enhancing the affinity of therapeutic T cell receptors (TCR) without altering their specificity is a significant challenge for adoptive immunotherapy. Current efforts have primarily relied on empirical approaches. Here, we used structural analyses to identify a glycine-serine variation in the TCR that modulates antigen sensitivity. A G at position 107 within the CDR3β stalk is encoded within a single mouse and human TCR, TRBV13-2 and TRBV12-5 respectively. Most TCR bear a S107. The S hydroxymethyl side chain intercalates into the core of the CDR3β loop, stabilizing it. G107 TRBV possess a gap in their CDR3β where this S hydroxymethyl moiety would fit. We predicted based on modeling and molecular dynamics simulations that a G107S substitution would increase CDR3β stability and thereby augment receptor sensitivity. Experimentally, a G107S replacement led to an ∼10–1000 fold enhanced antigen sensitivity in 3 of 4 TRBV13-2^+^ TCR tested. Analysis of fine specificity indicated a preserved binding orientation. These results support the feasibility of developing high affinity antigen specific TCR for therapeutic purposes through the identification and manipulation of critical framework residues. They further indicate that amino acid variations within TRBV not directly involved in ligand contact can program TCR sensitivity, and suggest a role for CDR3 stability in this programming.

## Introduction

T cells endowed with new specificities by T cell receptor (TCR) transduction have shown promise in cancer and other diseases [1]–[3]. Inadequate affinity may limit the activity of introduced TCR, and engineering enhanced responsiveness to peptide MHC (pMHC) ligand is an important challenge [4].

Affinity-enhancement has generally involved empirical approaches, such as *in vitro* selection after random mutagenesis [5]–[10]. Significantly, TCR binding to pMHC primarily results from contact associations with MHC rather than peptide antigen [11], [12]. Random mutations that increase TCR affinity will therefore often non-selectively increase affinity for MHC. Indeed, T cells modified with TCR mutated and selected for high affinity have been found to lose Ag specificity, responding to APCs alone [5], [8], [13]. It would be anticipated that mutant TCR with smaller affinity increases will likewise possess some increased reactivity to MHC. This may convert subthreshold engagements with self or other Ags into productive responses. Rational design, by using known TCR structures to direct mutations to residues less likely to alter Ag selectivity, may be a useful alternative to empirical approaches to modulate TCR affinity.

We and others observed a disproportionate representation of TRBV13-2^+^ TCR in myelin oligodendrocyte glycoprotein (MOG)-induced experimental allergic encephalomyelitis (EAE). Preferential TRBV13-2 use has also been seen in several other autoimmune diseases in mice and responses to some antigens, and TRBV13-2 is present on ∼50% of the NK-T cell repertoire [14]–[24]. Biased TRBV use need not indicate a clonal or oligoclonal response, but may be associated with heterogeneous TRAV and CDR3β sequences, a feature we identified after sequencing TCR from MOG-specific T cell clones [25], [26]. Bias may arise because specific V regions' CDRs have a predilection for specific Ags or binding orientations on MHC molecules [12], [27], [28].

To better understand structural differences that may underlie the preferential use of TRBV13-2 TCR, we aligned its sequence with that of other TRBV. We observed that most CDR3β incorporate a conserved N-terminal CASS motif in both mice (18/23 TRBV sequences) and humans (45/54) (Supp. Table S1). TRBV13-2 in mice and TRBV12-5 in humans were exceptions. These, unique to their species, bear a CASG motif. The S/G residues are buried within the CDR3β structure, not surface exposed. Structural studies demonstrated that a G107 leaves a gap in the CDR3β core, which we hypothesized would destabilize this critical antigen recognition domain. We predicted that a G107S substitution in TRBV13-2 TCR, would stabilize the CDR3β loop in configurations that retain antigen specificity, and could thereby increase TCR affinity for cognate ligand.

## Methods

### Ethics Statement

Studies were approved by and followed guidelines of the St. Jude Children's Research Hospital Animal Care and Use Committee (protocol 338).

### Mice

C57BL/6J (B6), B10.BR, and NOD/ShiLtJ mice were obtained from The Jackson Laboratory (Bar Harbor, ME), and B6.129-*H2^dlAb1-Ea^*/J (class II^−/−^) mice from Dr. P. Doherty (St. Jude Children's Research Hospital, Memphis, TN).

### TCR structures

TCR amino acid assignment is per International Immunogenetics Information System (IMGT) conventions (imgt.cines.fr). TCR structures were aligned, H-bonds calculated, and interatomic distances measured using Swiss PDB viewer v3.7. Images were produced and surfaces calculated using the PyMol molecular graphics system v1.1r1.

### Molecular Dynamics

The αβ TCR was extracted from the crystal structure of mouse 172.10 TCR complexed with pMHC (PDB ID: 1U3H). The β chain G107 was mutated to S with Swiss-Pdb viewer v3.7. Both the wt and the G107S structure complex were used as the starting points for molecular dynamics using Amber v10 software. The GB solvent model was used to mimic the solvent effect implicitly. Model and parameters were as described [29]. Amber FF03 force field [30] was used in all calculations, which consisted of 3 steps: a. 10000 cycles of energy minimization; b. 50 picoseconds of equilibration at constant volume by coupling the system to the Berendsen Heat bath with a constant temperature of 300K (canonical ensemble); c. 4.5 ns of molecular dynamics. The simulation setups were exactly the same for the WT and mutant complex. The RMSD values and potential energy values were obtained with Amber analysis tools. The covariance matrix method [31] was used to calculate entropies of the β chain CDR3 loop [^104^CAS(G/S)DAGGGYEQYFGP^119^]; the set of β chain CDR3 loop backbone N, C, Cα atoms were included in the entropy calculations.

### Peptides, Antibodies, and Flow Cytometry

MOG_35–55_ (MEVGWYRSPFSRVVHLYRNGK), alanine substitutions of this, GAD_206–220_, (TYEIAPVFVLLEYVT), and HEL_48–62_ (DGSTDYGILQINSRW) were synthesized and HPLC purified by the St. Jude Hartwell Center. Monoclonal antibodies (mAbs) specific for CD4 (clone H129.19) and TCR Vβ8.1, 8.2 (clone MR5-2) were from BD Biosciences. Flow cytometry was performed on a FACSCalibur (BD Biosciences), and flow cytometric sorting on a MoFlo high-speed cell sorter (DakoCytomation).

### TCR constructs and mutagenesis

The 1MOG9, 1 MOG244.2 [25], 3A9 [32], and PA19.5E11 [33], TCR α and β chains were isolated by PCR and cloned into the MSCV-I-GFP murine stem cell virus-based retroviral vector as described [25]. Site-directed mutagenesis was performed using the Quickchange II Site Directed Mutagenesis kit (Stratagene) and confirmed by DNA sequencing.

### Retroviral transduction

Retrovirus was produced as described [5] and used to infect surface TCR-deficient 4G4 or 4G4.CD4 T hybridoma cells. Transduced cells were flow cytometrically sorted on day 4 or 5 for TCR expression.

### Cytokine analysis

Transduced T cells were purified by flow cytometric sorting and expanded. Cells were cultured at 5×10^4^ per well with 3×10^5^ 3000-rad irradiated APCs and the indicated stimulus. Culture supernatant was collected at 24 h and analyzed for IL-2 by sandwich ELISA (BD Pharmingen) or Bio-Plex (Bio-Rad) assay. Samples were analyzed in triplicate.

## Results

### Comparative analysis of CDR3β in TRBV13-2 and other TCR

**Figure 1:**
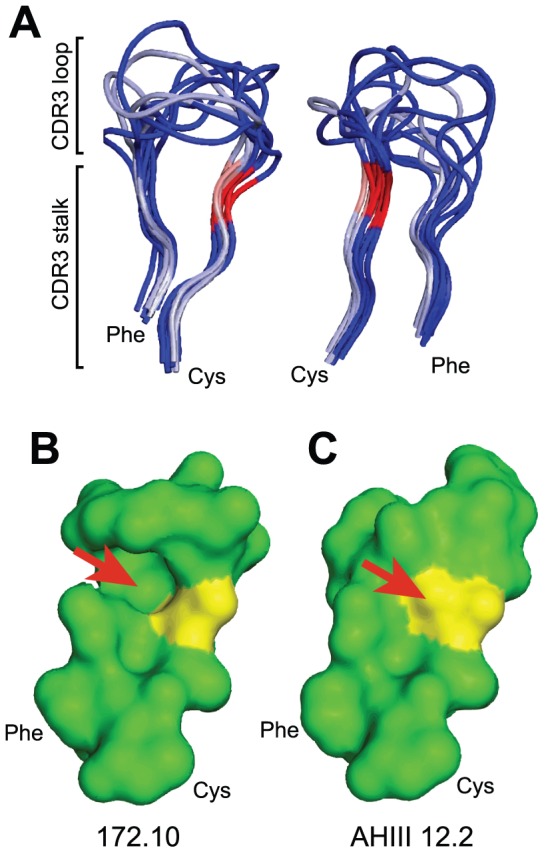
Impact of a G versus S residue at TCRβ position 107. (A) Three TRBV13-2 (silver) and 7 non-TRBV13-2 (blue) TCRαβ structures (listed in the text) were aligned. Their CDR3β peptidyl backbones are displayed in two orientations, indicating highly conserved stem structures. G107 residues are in pink and S107 in red. The structural impact of a G107 versus S107 is exemplified in the mouse TRBV13-2^+^ 172.10 TCR (B) and TRBV13-3^+^ AHIII 12.2 TCR (C). The arrow indicates the location of the S107 side chain in AHIII 12.2 that is absent in the 172.10 TCR.

The N-terminal sequence of TRBV13-2^+^ CDR3β in mice and TRBV12-5^+^ CDR3β in humans is ^104^CAS**G**. This contrasts with the ^104^CAS**S** sequence in the majority of TRBV loci (78% in mice, 83% in humans; Supp. Table S1 and Supp. Fig. S1). Coordinates for three TRBV bearing the CASG motif and seven with the CASS motif were aligned to define the molecular consequences of this G/S variation. Overlay plots of the TCR, highlighting just the CDR3β showed tight juxtaposition of the peptidyl backbones of the CDR3 stalks, which includes the G/S variant residue (Fig. 1a). This was true regardless of TCR source, human or mouse, or the alternative presence of G or S at position 107. In contrast to the stalks, backbones of the surface exposed CDR3β loops showed greater variability.

**Figure 2:**
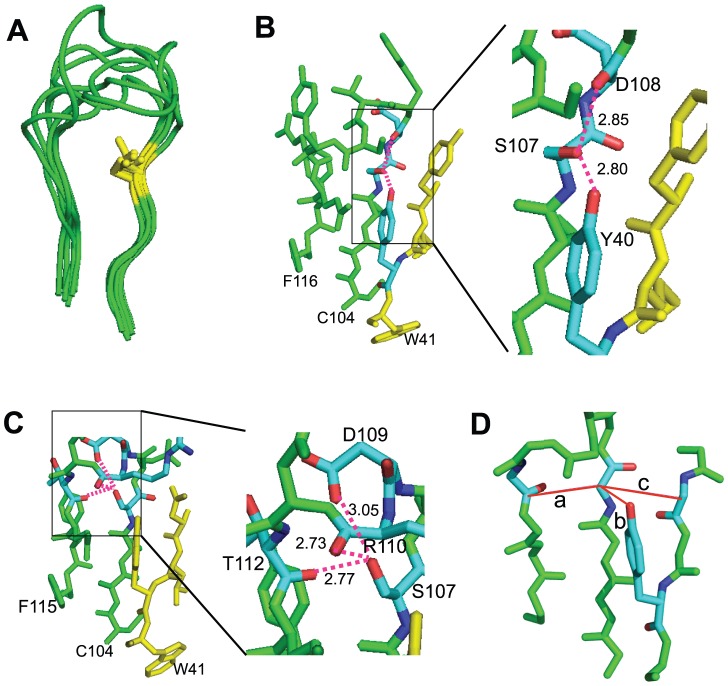
S107 hydroxymethyl positioning within the CDR3β loop. (A) Positions of the S107 hydroxymethyl side chains (yellow) for 7 overlayed TCR structures. (B) The S107 hydroxyl in the AHIII12.2 TCR is oriented outside of the plane of the CDR3β loop, permitting H-bonding with the backbone carboxyl O of D108 and the side chain hydroxyl of Y40. H-bonds are indicated by dashed lines, and distances by numbers in Å. (C) The S107 hydroxyl in the KK50.4 TCR is within the plane of the CDR3β loop, permitting H-bonding with the backbone carboxyl O of R110, the D109 side chain, and carboxyl oxygen of T112. (D) Distances were measured between the α carbon of the S or G at 107 and the backbone carbonyl carbon of the most proximate amino acid in the countercurrent strand of the CDR3β (distance a), the Y40 O if present (distance b), and the α carbon of TRBV residue 42 (distance c). The diagram, derived from the KK50.4 TCR, shows peptidyl backbone only with the exception of the Y40 side chain.

Two consistent differences were attributable to the G/S variation. First, a G107 left a gap within the center of the CDR3β loop (Fig. 1b, TRBV13-2^+^ 172.10 TCR; see Supp. Fig. S2a for other structures). When an S was present, its hydroxymethyl side chain was oriented to intercalate into this space, thereby generating van der Waals interactions with other residues (Fig. 1c, TRBV13-3^+^ AHIII 12.2 TCR; Supp. Fig. S2b). Second, for 6/7 of the CASS structures, the S107 hydroxyl moiety was oriented outside of the plane of the CDR3 loop (Fig. 2a). This facilitated hydrogen bonding with backbone CDR3β residues upstream from the S residue and, when present, a Y40 side chain occupying a TRBV β-sheet strand juxtaposed to the CDR3 (Fig. 2b; AHIII 12.2 TCR). For 1/7 CASS TCR, KK50.4, the S107 hydroxymethyl was oriented in the plane of the CDR3, forming H bonds with both immediately upstream and countercurrent residues of the loop (Fig. 2c). Therefore S107 bonds both through van der Waals interactions and H-bonding with adjacent residues. This would be anticipated to stabilize CASS but not CASG CDR3β structures.

### Absence of structural accommodation to G107

It was possible that CASG TCR had an altered configuration compared with CASS TCR, compensating for the space vacated by the S107 side chain. This was however not apparent. Distance between the TRBV G107 or S107 backbone α carbon and the most proximate TRBJ backbone carbonyl carbon on the opposing CDR3 strand were not significantly different regardless of whether a G or S was present at position 107 (Fig. 2d distance a and Table 1). This was likewise true for distances between the 107 backbone carbon and that of the Y40 side chain oxygen (Fig. 2d, distance b) or the backbone α carbon of the opposed beta sheet residue 2 amino acids upstream of this Y (Fig. 2d, distance c). The single exception among the tight clustering of interatomic distances among the TCR was the CASS-containing, myelin basic protein-specific, 3A6 TCR, which showed increased interatomic distances (Table 1). This, together with the atypical structural gap above the S107 position of the 3A6 CDR3β (Supp. Fig. S2b), argues for a distinct orientation of this autoreactive CDR3β loop. Overall, however, the data indicates that crystallographically-determined TCR spatial configuration is not significantly impacted by a G versus an S residue at 107.

**Table 1:** Interatomic distances in G107 or S107 TCR.

TCR	TRBV13-2	PDB ID	CDR3β	Distance a	Distance b	Distance c
D10	Y	1D9K	CASGGQGRAEQFF	4.81	5.73	5.40
172.10	Y	1U3H	CASGDAGGGYEQYF	4.98	5.28	4.90
2C	Y	2OI9	CASGGGGTLYF	5.12	5.25	4.85
*Mean ± S.D.*				*4.97±0.16*	*5.42±0.27*	*5.05±0.30*
HA1.7	N	1FYT	CASSSTGLPYGYTF	5.06	NA	4.82
AHIII 12.2	N	1LP9	CASSDWVSYEQYF	5.17	4.95	4.88
N15	N	1NFD	CASSLRWGDEQYF	4.55	NA	5.26
JM22	N	1OGA	CASSRSSYEQYF	4.59	6.04	4.96
1G4	N	2BNQ	CASSYVGNTGELFF	4.82	NA	4.98
KK50.4	N	2ESV	CASSQDRDTQYF	4.84	5.67	4.93
3A6	N	1ZGL	CASSLADRVNTEAFF	6.12	NA	5.60
*Mean ± S.D.*				*5.02±0.53*	*5.55±0.55*	*5.06±0.28*

TCR with a G107 (TRBV13-2^+^) or S107 (TRBV13-2^−^) TCRβ were analyzed. Distances measured are indicated in Fig. 2d and the text, and are measured in Å. PDB ID and CDR3β sequence of structures studied are listed.

**Figure 3:**
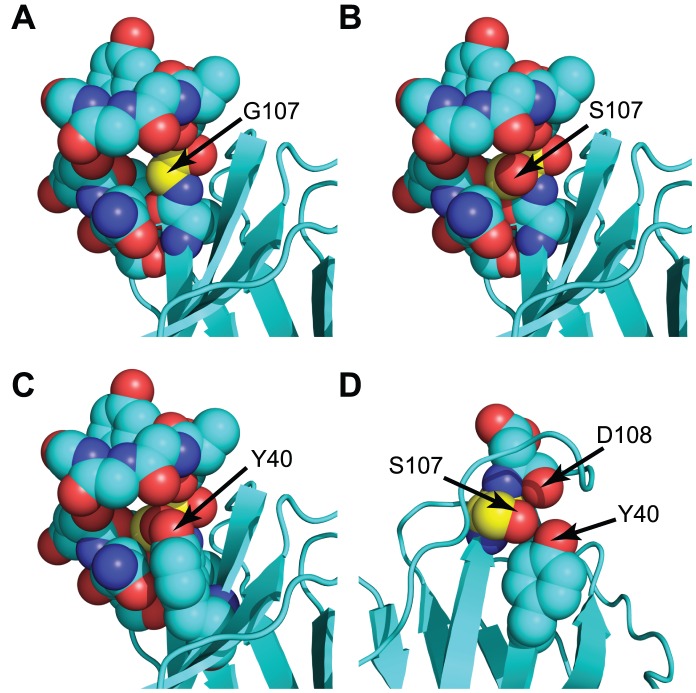
Modeling of a G107S mutation in the 172.10 TCR. (A) Arrow and yellow colored carbon atoms indicate the position of G107 within 172.10. A gap is noticeable within the center of the CDR3β. (B) A G107S substitution, modeled without altering the atomic positions of other amino acids, demonstrates the potential for the S hydroxymethyl to fill the gap. (C) Addition of the Y40 side chain to the structure in (B) using coordinates from the original 172.10 structure further shows accommodation of the G107S substitution. (D) Removal of side chains of select amino acids and rotation of the structure relative to (C) demonstrates the juxtaposition and potential for H bonding between the inserted S107 hydroxyl, the D108 carboxyl O, and the Y40 side chain hydroxyphenyl in a manner corresponding to that observed in the AHIII 12.2 and other TRBV13-2^−^ TCR crystal structures (see Fig. 2b).

The failure of G107 TCR to accommodate for the absence of a hydroxymethyl side chain suggested that a G107S substitution should fit within the CDR3β in TRBV13-2^+^ TCR. Modeling of G107S mutations indeed showed that the solved 172.10, D10, and 2C crystal structures could contain the additional hydroxymethyl group in orientations equivalent to that seen in the CASS TCR without modifying any other atomic positions (Fig. 3a–d and not shown). Therefore, a gap is left in G107 TCR that is able to hold an S107 side chain. This gap would be anticipated to destabilize the CDR3β. A G107S substitution would be expected to stabilize the CDR3β structure by H-bonding and van der Waals interactions with neighboring residues, and if able to maintain a conformation favorable for pMHC interactions should increase TCR reactivity.

### Enhanced responsiveness of G107S TCR

To test the impact of G107S substitutions on TRBV13-2^+^ TCR, we generated these in 2 MOG specific TCR we previously cloned, 1MOG9 and 1MOG244.2 [25]. These TCR bear CDR3 identical in length to the crystallized TRBV13-2^+^ 172.10 TCR described above. Sequence was also similar; only a single amino acid differed between the CDR3β of 172.10 and 1MOG244.2 and 5 amino acids differed with 1MOG9 (172.10: CASGDAGGGYEQYF; 1MOG9: CASGD**W**GG**EDTL**YF; 1MOG244.2: CASGDAG**T**GYEQYF). Considering the conserved structures of TCR V regions in general and the identical length and nearly identical sequences of these TCRβ, their frameworks should be similar.

We transduced retrovirus incorporating wt or G107S 1MOG9 or 1MOG244.2 TCR into TCRαβ-deficient 4G4.CD4 T cell hybridomas. Cells were sorted for similar TCR expression levels and stimulated with titrations of MOG_35–55_ peptide. Because the wt and G107S TCR-transduced 4G4.CD4 cells were essentially identical except for the single amino acid difference in the CDR3β at position 107, response sensitivity to MOG_35–55_ should primarily reflect TCR binding affinity.

**Figure 4:**
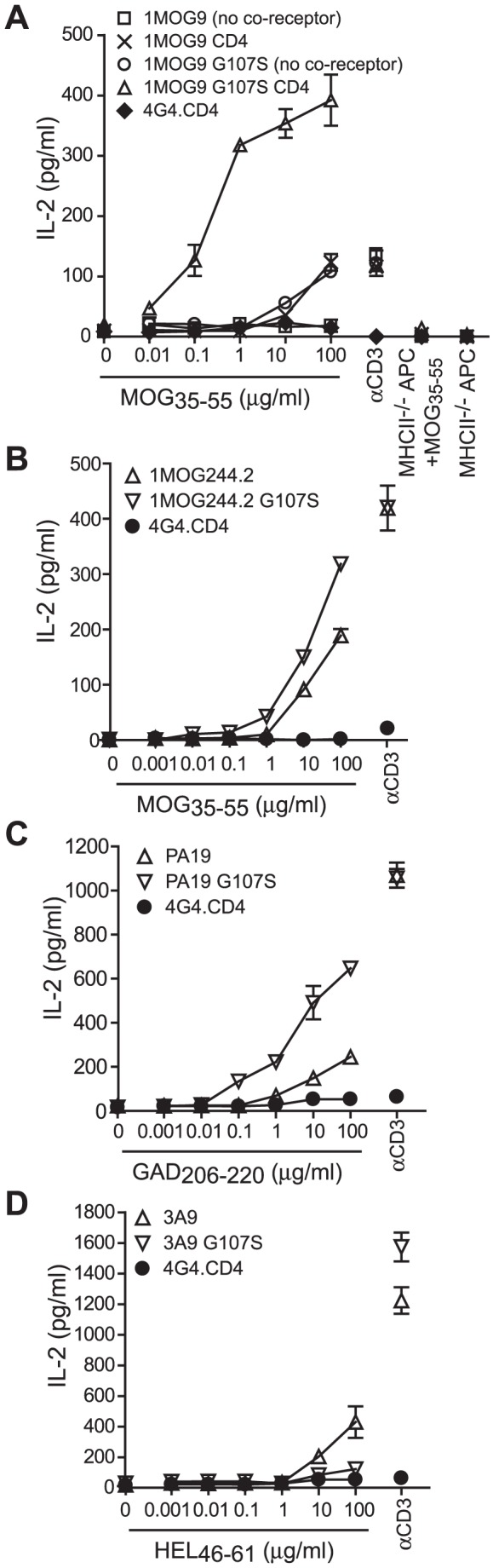
Enhanced antigen sensitivity mediated by a G107S substitution. (A) The 1MOG9 wt or G107S TCR were transduced into 4G4 or 4G4.CD4 TCRαβ-deficient hybridoma cells. The cell lines were flow cytometrically sorted for equivalent TCR expression and stimulated with MOG_35–55_ peptide or αCD3 and irradiated C57BL/6 or class II^−/−^ splenic APCs as indicated. IL-2 production was measured at 24 h. Similar studies were performed with 4G4.CD4 cells transduced with wt or G107S substituted 1MOG244.2 (B), PA-19 (C), and 3A9 TCR (D).

The 1MOG9 G107S TCR dramatically increased MOG_35–55_ sensitivity, ∼1000 fold, and substantially increased maximal response (Fig. 4a). This did not result from acquisition of antigen-independent responsiveness, as neither wt nor G107S TCR-transduced cells responded to APC in the absence of antigen. Further, response remained class II MHC restricted as neither cell type responded to antigen in the presence of class II MHC^−/−^ APC. Therefore the addition of a single hydroxymethyl moiety within the center of the CDR3β loop can dramatically alter TCR sensitivity.

The presence of CD4 co-receptor boosts TCR signaling by recruiting the downstream kinase lck and, to a lesser extent, by its adherence to class II MHC [34]. To test whether increased antigen sensitivity of G107S TCR led to co-receptor independence, we also transduced wild type or G107S 1MOG9 receptor into 4G4 T cells lacking CD4 or CD8 co-receptor. Whereas no response was detected among the 1MOG9 T cells at any concentration of Ag, response was seen with the 1MOG9 G107S mutant, though this was diminished ∼3log10 compared with transductants expressing CD4 (Fig. 4a).

Analysis of the 1MOG244.2 TCR also demonstrated increased sensitivity with the G107S substitution. This, however, was more moderate, with a 5–10 fold increase (Fig. 4b). To determine if increased antigen responsiveness could also be seen with TCR specific for other antigens, we generated similar substitutions in two additional TRBV13-2^+^ TCR, the glutamic acid decarboxylase (GAD)_206–220_/IA^g7^-specific PA19 and hen egg lysozyme (HEL)_46–61_/IA^k^-specific 3A9 receptors. The presence of an S107 increased antigen sensitivity ∼100 fold in the PA19 TCR whereas it abolished recognition in the 3A9 TCR (Fig. 4c, d). Therefore, consistent with structural predictions, the addition of a hydroxymethyl moiety to G107 can increase antigen sensitivity among some TCR. Impressively, 3 of 4 analyzed TCR displayed an increase in antigen sensitivity of ∼10–1000 fold. The loss of reactivity in 1/4 of the TCR is not surprising as conformational limitations imposed by the S107 may conceivably hinder recognition.

### Specificity of S at position 107

**Figure 5:**
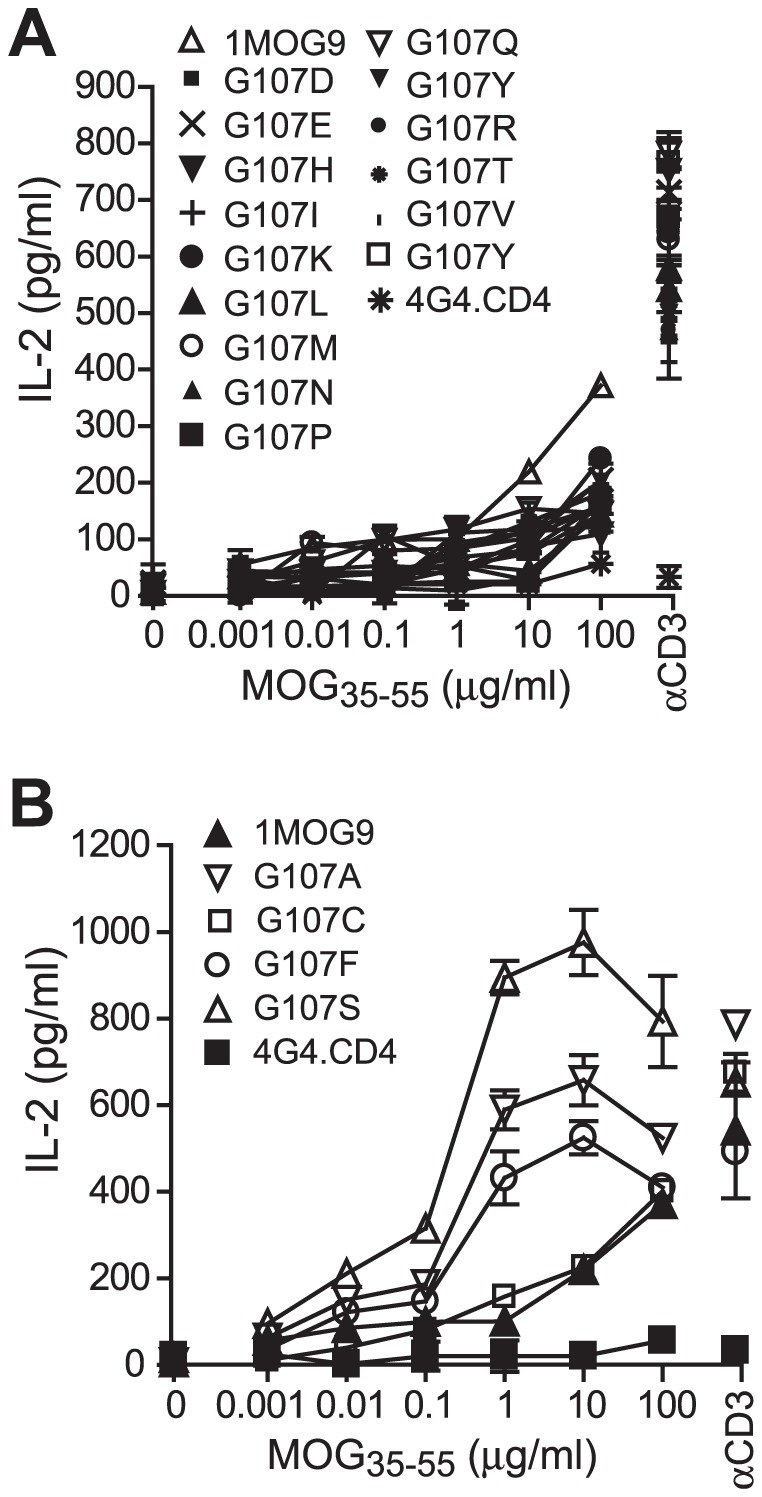
Selectivity of the G107S mutation. 1MOG9 TCR was mutated to insert all other amino acids at position 107. TCR-transduced 4G4.CD4 cells, sorted for equivalent TCR expression, were stimulated with titrations of MOG_35–55_. (A) TCR with amino acid substitutions leading to a diminished (A) or enhanced or similar (B) response compared with the wt 1MOG9 are shown.

Considering the relatively conserved use of a CASS motif in CDR3β, we anticipated that the increased antigen sensitivity endowed by the S substitution would be specific to this residue. To test this, we substituted the remaining 18 amino acids at the G107 position in the 1MOG9 TCR. Transduced 4G4.CD4 cells were again sorted for similar TCR expression levels (Supp. Fig. S3). Of the substituted TCR, 15 diminished antigen sensitivity when compared with wt TCR, or abolished recognition (Fig. 5a). A G107C substitution showed sensitivity similar to that of the wild type receptor (Fig. 5b). An A and to a lesser extent F substitution increased antigen sensitivity ∼100 fold, substantial though to a less than the ∼1000 fold increase with the S substitution. Structural modeling of the G107A suggested that it would situate similarly to the S, though it lacks the S hydroxyl. In contrast, the enhanced sensitivity of the bulky hydrophobic F substitution undoubtedly occurs through structurally distinct interactions. Therefore, few amino acid changes at position 107 can enhance TCR response, and the conserved S present in most CDR3β is specifically well suited for this location.

### Fine specificity of TCR recognition

**Figure 6:**
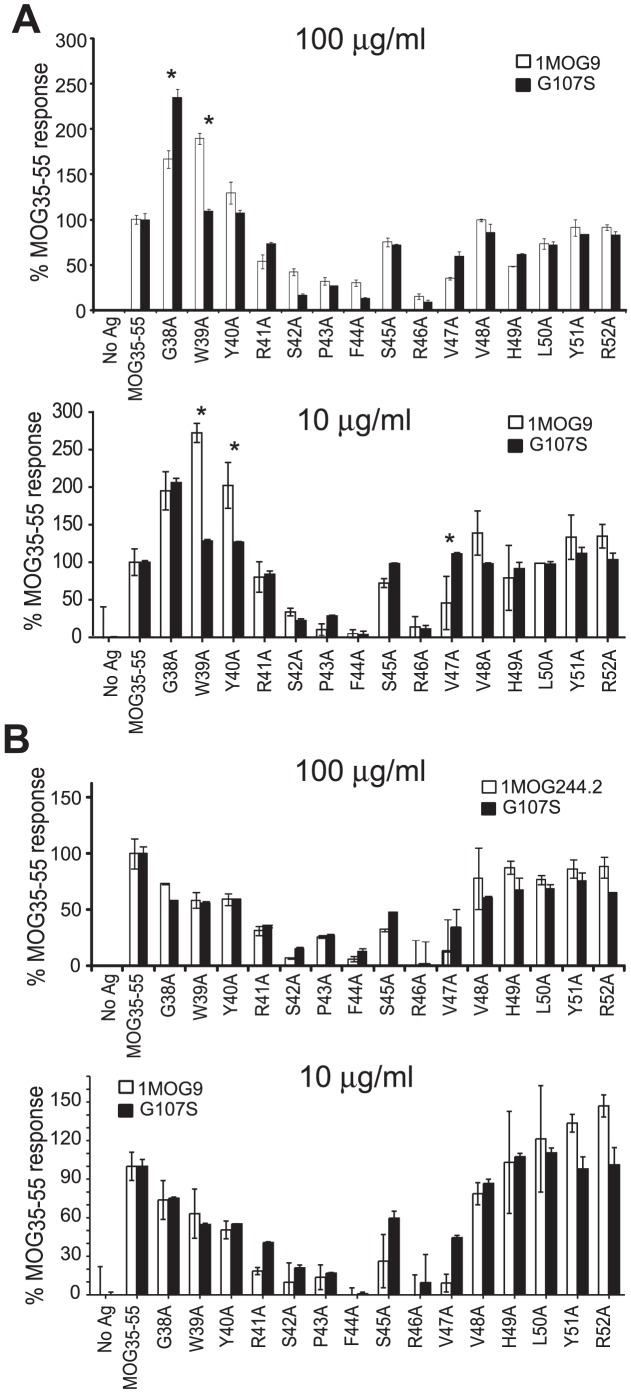
Fine specificity of wt and G107S TCR. To determine if the G107S substitution influenced fine specificity, 1MOG9 (A) or 1MOG244.2 (B) transduced 4G4.CD4 cells or their corresponding G107S mutants were stimulated with 10 or 100 µg/ml of the indicated A-substituted MOG_35–55_ peptides. IL-2 production, normalized to the response to wt MOG_35–55_, is plotted. Plots are representative of 3 independent studies performed with 10 µg/ml and 2 with 100 µg/ml peptide Ag. *, p<0.05 by ANOVA.

Our modeling showed that a G107S substitution can fit within the center of a CDR3β without substantially altering its overall structure, suggesting that a G107S mutation should not prominently impact fine specificity of pMHC recognition. To test this, we assessed IL-2 production against a panel of 15 ala-substituted MOG_35–55_ peptides by 1MOG9 or 1MOG244.2 and the equivalent G107S-modified T cells. Data was normalized against the response to unmodified peptide. The overall response pattern of the TCR was similar. Ala substitutions in S42, P43, F44, and R46 strongly diminished responses by both the 1MOG9 and 1MOG244.2 TCR (Fig. 6a, b). No difference in the wt and G107S modified TCR using either high or low concentrations of Ag was observed for these critical residues. Some differences were observed with other ala substitutions. Specifically, for the 1MOG9 TCR and its G107S mutant, responses differed to G38A and W39A substitutions after stimulating with 100 µg/ml peptide, and W39A, Y40A, and V47A with 10 µg/ml peptide. However, of these only W39A remained significantly different in repeat experiments, and only in one of two additional analyses at 10 µg/ml (data not shown). In contrast to the 1MOG9 TCR, no significant differences were observed when comparing the 1MOG244.2 wt and G107S responses to any of the substituted peptides. Therefore the G107S mutation enhances sensitivity in the MOG-specific TCR. However, consistent differences in response to ala-substituted peptides were not reliably obtained and response to the MOG_35–55_ residues critically required for TCR response did not differ. This indicates that core recognition is preserved when comparing the wt and G107S TCR.

## Discussion

Greater than 20 and 50 TRBV are available in mice and humans respectively, yet specific immune responses may rely on just one or a few of these [27]. G107 is unique to TRBV13-2 in mice and TRBV12-5 in humans. In each species, other TRBV dominantly express a S at this location. As the hydroxymethyl side chain of the more common S is positioned to intercalate into the core of the CDR3β loop, we reasoned that alternative amino acids at that position should distort the CDR3β's conformation and thereby influence pMHC recognition. Surprisingly, our evaluation of previously characterized TCR structures indicated that the absence of a S107 side chain in TRBV13-2^+^ TCR did not lead to substantive structural alterations. TRBV13-2^+^ and other TCR were similar, but a gap was left in TRBV13-2^+^ receptors where a S hydroxymethyl would otherwise fit. Modeling indicated that TRBV13-2^+^ TCR could accommodate a G→S substitution without displacement of any other residue, indicating that the bonding provided by the S107 hydroxymethyl is not essential to preserve CDR3β structure. We predicted that the gap present in TRBV13-2 CDR3β will be energetically unfavorable, and that a G107S replacement will increase the stability and hence affinity of TRBV13-2^+^ TCR. This was born out in our experimental findings, as 3 of 4 TRBV13-2^+^ TCR showed increased responsiveness after G107S modification.

Biophysical analyses of G107S TCR will be needed to fully evaluate the mechanism of their enhanced reactivity. We can however speculate on the structural impact of the substitution. The free energy of TCR binding to pMHC will be proportional to the change in enthalpy, or chemical heat, associated with their interaction, and inversely proportional to the change in entropy, or randomness, in the molecules. CDR flexibility, which will be related to entropy, is a fundamental element of current models of TCR–pMHC engagement, allowing TCR to optimally conform to the surface of its ligand. Indeed, molecular accommodation was apparent in solved structures of individual TCR bound to different pMHC complexes or when comparing free and pMHC-bound TCR [11]. An inverse relationship has also been observed between TCR avidity and degeneracy, indicating that as TCR gain flexibility and an increasing ability to bind different ligands they also lose binding affinity [35], [36]. A single report has specifically examined the motion within a single chain TCR using NMR spectroscopy [37]. Interestingly, this identified enhanced CDR3 motility in the TRBV13-2^+^ D10 TCR.

Studies with antibodies have more clearly demonstrated the role of entropy in modulating binding affinity. In some cases, affinity is heavily governed by entropic features. Specific residue changes, such as occurs with affinity maturation, may enhance affinity by modulating receptor entropy [38]–[40]. Therefore, molecular alterations that influence CDR entropy may also affect affinity. The G107S substitution in TCR assessed here, by stabilizing the CDR3β loop through H-bonding and van der Waals interactions would be expected to diminish receptor entropy. This may enhance affinity if it is not superseded by opposing enthalpic changes.

**Figure 7:**
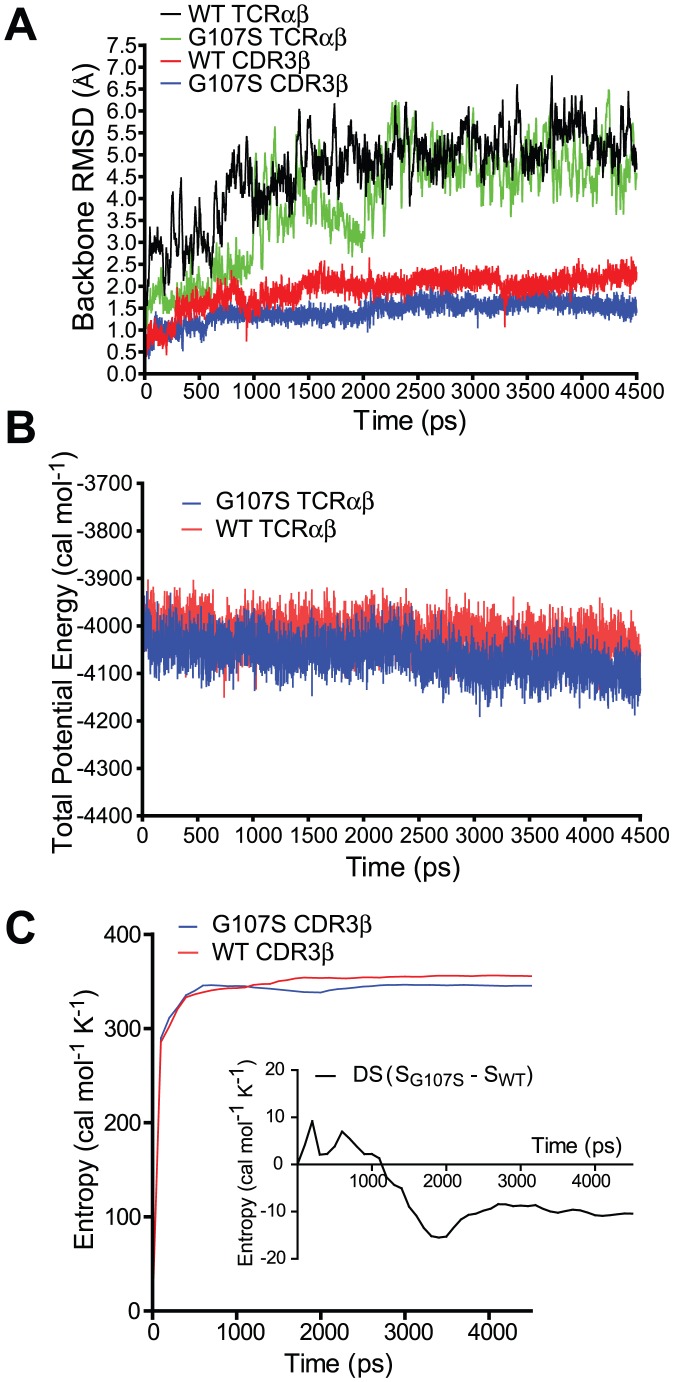
Molecular Dynamics analysis of a G107S mutation. WT or G107S mutant 172.10 TCR underwent 4.5 ns of MD. (A) RMSD from initial structure of the TCRαβ or the CDR3β is plotted versus time of MD. (B) Potential energies, an indicator of overall structural stability, of TCRαβ structures during the course of MD is plotted. (C) Entropic analysis of the CDR3β backbone is shown. Inset demonstrates the difference in entropy between wt and mutant CDR3β.

As an initial assessment of the impact of a G107S substitution on TCR entropy, we analyzed the molecular dynamics (MD) of TRBV13-2 CDR3β in silico (Fig. 7). The crystallographically defined 172.10 TCRαβ structure without pMHC ligand and with or without a G107S substitution underwent initial energy minimization followed by 4.5 ns MD in an implicit solvent model using Amber software. Structures stabilized after ∼2.5 ns. The G107S mutant displayed overall preserved structural integrity, and indeed both the entire TCR structure and the CDR3β peptidyl backbone had a lower root backbone mean square deviation (RMSD) from the initial minimized structure than the wt TCR (Fig. 7a). Both the wt and G107S TCR showed similar overall stabilities (Fig. 7b). More interestingly, the G107S CDR3β showed diminished motility compared with the wt, corresponding to a decrease in the mutant's entropy (Fig. 7c). Although the change in entropy was a small percent of total entropy, we can estimate that this magnitude of change may potentially lead to a >2log10 difference in binding affinity (not shown).

These MD results are therefore consistent with the hypothesis that a G107S substitution promotes TCR affinity by diminishing entropy. Certainly, though, entropic modulation will not be independent of enthalpic changes, and a full assessment of this will require direct binding measurements of the TCR in association with their ligands. Indeed, restrictions in CDR3β flexibility may exclude conformations enthalpically favorable for pMHC interaction, and could explain the loss of reactivity in the 3A9 TCR with a G107S mutation.

It is interesting to further speculate that G107 is also important in the increased use of TRBV13-2 in NK-T cells and specific autoimmune models, for instance by enhancing flexibility and degeneracy of these TCR. Similar concepts have been raised and debated previously [41], [42]. Our findings may indicate relevance of the G107 in the preferential use of this TCR. However, it would seem unlikely that the variation at position 107 is an exclusive control point in establishing response thresholds among different TCR. Rather, other amino acid variations in proximity to the CDR3 would be expected to similarly influence TCR responsiveness.

Importantly, the ability to modulate TCR affinity is of considerable therapeutic concern. Genetically modified T cells transduced with TCR specific for tumor antigens have shown efficacy in clinical trials, which are being expanded to include mutated TCR [5], [6], [13], [43]. TCR modified T cells may have use in cancer, autoimmunity, and infection. We and others have shown that such mutated TCR may also develop altered antigen specificity, particularly the acquisition of self-reactivity. Substituting a S in G107-bearing TRBV, which we hypothesize acts by stabilizing the CDR3β rather than through altered binding to MHC, may potentially boost overall sensitivity without substantially altering specificity. Indeed, we did not observe peptide-independent APC reactivity, or altered fine specificity with this modification. In summary, our results provide evidence for a genetic variation in a single amino acid in a non-pMHC contact residue of a TCR V gene controlling TCR sensitivity for antigen, and support a new approach to generate high affinity TCR for therapeutic purposes through modification of G107^+^ TCR.

## Supporting Information

Figure S1 **Location of G/S107 variation in CDR3.** Structural model of the 172.10 TCR (PDB ID: 1U3H) demonstrating the CDR3β (yellow) associating with MHC-bound peptide (red). The TCRβ chain is in green and α chain in purple. The position of the G/S variation is in pink.
(EPS)

Figure S2 **CDR3β structures.** Aligned structures of characterized CDR3β loops from TCR with a G107 are shown in (A) and with an S107 in (B). The G or S107 residues are colored yellow. The position of the S107 hydroxymethyl side chain, or the equivalent position for TCR bearing a G107, is indicated by the arrow.
(EPS)

Figure S3 **TCR expression in TCR transduced 4G4.CD4 T cells.** 4G4.CD4 T cells were transduced with the 1MOG9 or indicated mutant TCR, expanded, and flow cytometrically sorted for TCR expression. TCR expression levels for untransduced 4G4.CD4 cells or for the indicated transduced lines is shown.
(EPS)

Table S1 **Aligned sequences of the V region of TCR CDR3β.** Sequences of the C-termini of human and mouse TRBV identified using the Immunogentics Information System (imgt.cines.fr) are indicated, and begin at the conserved C at position 104. A S at position 107 is indicated by a red color, and a G by a green color. The majority of mouse and human TRBV have a CASS motif at this site.
(DOC)
