# Supplementary material for: Rational Design of T Cell Receptors with Enhanced Sensitivity for Antigen
Source: PLoS One. 2011 Mar 23;6(3):e18027. doi: 10.1371/journal.pone.0018027 (PMC3063236; doi:10.1371/journal.pone.0018027)
Supplement: Table S1 — Aligned sequences of the V region of TCR CDR3β. Sequences of the C-termini of human and mouse TRBV identified using the Immunogentics Information System (imgt.cines.fr) are indicated, and begin at the conserved C at position 104. A S at position 107 is indicated by a red color, and a G by a green color. The majority of mouse and human TRBV have a CASS motif at this site. (DOC) [file pone.0018027.s004.doc]

Mouse TRBV

TRBV1 CTCSA

TRBV2 CASSQ

TRBV3 CASSL

TRBV4 CASS

TRBV5 CASSQ

TRBV12-1 CASSL

TRBV12-2 CASSL

TRBV13-1 CASSD

TRBV13-2 CAS**G**D

TRBV13-3 CASSD

TRBV14 CASSF

TRBV15 CASSL

TRBV16 CASSL

TRBV17 CASSR

TRBV19 CASSI

TRBV20 CGAR

TRBV21 CASSQ

TRBV23 CSSSQ

TRBV24 CASSL

TRBV26 CASSL

TRBV29 CASSL

TRBV30 CSSR

TRBV31 CAWS

Human TRBV

TRBV2 CASSE

TRBV3-1 CASSQ

TRBV4-1 CASSQ

TRBV4-2 CASSQ

TRBV4-3 CASSQ

TRBV5-1 CASSL

TRBV5-3 CARSL

TRBV5-4 CASSL

TRBV5-5 CASSL

TRBV5-6 CASSL

TRBV5-7 CASSL

TRBV5-8 CASSL

TRBV6-1 CASSE

TRBV6-2 CASSY

TRBV6-3 CASSY

TRBV6-4 CASSD

TRBV6-5 CASSY

TRBV6-6 CASSY

TRBV6-7 CASSY

TRBV6-8 CASSY

TRBV6-9 CASSY

TRBV7-1 CASSS

TRBV7-2 CASSL

TRBV7-3 CASSL

TRBV7-4 CASSL

TRBV7-6 CASSL

TRBV7-7 CASSL

TRBV7-8 CASSL

TRBV7-9 CASSL

TRBV9 CASSV

TRBV10-1 CASSE

TRBV10-2 CASSE

TRBV10-3 CAISE

TRBV11-1 CASSL

TRBV11-2 CASSL

TRBV11-3 CASSL

TRBV12-3 CASSL

TRBV12-4 CASSL

TRBV12-5 CAS**G**L

TRBV13 CASSL

TRBV14 CASSQ

TRBV15 CATSR

TRBV16 CASSQ

TRBV17 YSS**G**

TRBV18 CASSP

TRBV19 CASSI

TRBV20-1 CSAR

TRBV23-1 CASSQ

TRBV24-1 CATSDL

TRBV25-1 CASSE

TRBV27 CASSL

TRBV28 CASSL

TRBV29-1 CSVE

TRBV30 CAW
